# Heavy metals and metalloid levels in the tissues of yellow-legged gulls (*Larus michahellis*) from Spain: sex, age, and geographical location differences

**DOI:** 10.1007/s11356-022-19627-8

**Published:** 2022-03-17

**Authors:** Jorge Vizuete, David Hernández-Moreno, Ana López-Beceiro, Luis Eusebio Fidalgo, Francisco Soler, Marcos Pérez-López, María Prado Míguez-Santiyán

**Affiliations:** 1 Toxicology Area, Faculty of Veterinary Medicine (UEX), 10003 Caceres, Spain; 2 Department of Environment and Agronomy, National Institute of Agriculture and Food Research and Technology (INIA), Spanish National Research Council (CSIC), Carretera de la Coruña Km 7, Madrid, Spain; 3 Department of Veterinary Clinical Sciences, Faculty of Veterinary Medicine (USC), 27003 Lugo, Spain; 4 IMPROCAR Research Institutes, Caceres, Spain; 5 INBIO G+C Research Institutes, Caceres, Spain

**Keywords:** Metals, Seabird, Bioaccumulation, Sentinel species, Feathers

## Abstract

In the present study, mercury (Hg), cadmium (Cd), lead (Pb), selenium (Se), and arsenic (As) were measured in liver, kidney, and feathers of adult, juvenile, and chick seagulls (*Larus michahellis*) collected from the northwest of Spain. Age, sex, and the geographical location of samples were considered variables that can influence metal bioaccumulation, for which concentrations were determined by means of ICP-MS. The mean concentrations (dry weight) found in seagulls were 7.01 ± 0.37 mg Hg/kg, 22.82 ± 2.83 mg Cd/kg, 7.36 ± 1.36 mg Pb/kg, 18.64 ± 0.63 mg Se/kg, and 10.64 ± 0.59 mg As/kg. Regarding the different factors analyzed, Hg was the only metal showing sex-related differences, being significantly higher (*p* < 0.05) the concentrations found in feathers of males (1.26 ± 0.12 mg/kg) than those in females (0.99 ± 0.11 mg/kg). A highly significant (*p* < 0.01) increase in levels of some metals was found in liver related to the increase of age: Hg (adults (A) 3.33 ± 0.22 mg/kg vs chicks (C) 1.76 ± 0.28 mg/kg), Cd (A 4.74 ± 0.62 mg/kg vs C 1.79 ± 0.2), Pb (A 0.65 ± 0.12 mg/kg vs juveniles 0.4 ± 0.11 mg/kg), and Se (A 7.56 ± 0.43 mg/kg vs C 5.24 ± 0.53 mg/kg). Positive correlations between Cd-Hg and Se–Hg were found in liver (*p* < 0.001), kidney (*p* < 0.001), and feathers (*p* < 0.05 and *p* < 0.001, respectively). The associations found may reflect antagonistic interactions between Se and Cd on Hg toxicity. The results suggest that *L. michahelli*s can reveal local contamination around the foraging and breeding sites and can be a very useful monitoring instrument for assessing heavy metal contamination and sentinel species of environmental health.

**Supplementary Information:**

The online version contains supplementary material available at 10.1007/s11356-022-19627-8.

## Introduction

The environment is exposed to different anthropogenic pollutants, such as metals, which can derive from several activities. Metals are natural components in the environment; many of them are essential for the organism (selenium (Se), phosphorus (P), zinc (Zn), copper (Cu)), whereas others are highly toxic or become toxic depending on the exposure concentration. Heavy metals such as lead (Pb), cadmium (Cd), and mercury (Hg) could come from human activities or natural sources such as erosion of rocks, wind-blowing dusts, volcanic activity, and forest fires. These metals have no biological functions, are not required by organisms, are highly toxic, may induce both acute and chronic toxicological effects, and have important ecotoxicological effects on wildlife and human health. In addition, they biomagnify, accumulate, and persist in the environment and in living organisms, ascending the different trophic levels that make up an ecosystem through the food chain (Merian 1991). Therefore, exposure to these chemicals is of particular concern. The transfer of pollutants from the environment to biota is influenced by different environmental and biological parameters (Baker et al. 2003). The list of heavy metals with greater presence in the sediment of Galician estuaries (Spain) includes the following: Hg, Cd, Pb, nickel, Zn, manganese, chromium, Cu, and cobalt (Cobelo-Garcıa and Prego 2003).

Organisms living in wetland systems can bioaccumulate organic and certain inorganic substances over time and are at risk for both lethal and sub-lethal effects, as their body burdens increase (Gochfeld 1997). Studying wildlife populations in such environments provides relevant information about the viability and balance of those ecosystems. The usefulness of studying birds as bioindicators of environmental contamination has been recognized in many research projects because these animals occupy different trophic levels in ecosystems, are widely distributed, and are sensitive to atmospheric changes in the environment (Egwumah et al. 2017). Specifically, waterbirds may serve as sentinel species, since pollutants can enter avian organisms through several routes (dermic, inhalation, ingestion), thus allowing monitoring of the health status of the ecosystem where they live (Zhang and Ma 2011). Seagulls are bird species potentially used as bioindicators (Burger and Gochfeld 2000). Indeed, since 1971, the herring gull (*L. argentatus*) has been used as a sentinel species for monitoring the levels of different elements around the world (Koster et al. 1996; Leonzio et al. 1986; Savinov et al. 2003).

The yellow-legged gulls (*L. michahellis*), a seagull species, represents an abundant population in the Cantabric Sea that has increased by almost 150% during the last three decades (Arizaga et al. 2009). This kind of gull can reach 1.5 kg weight and lives mainly on rocky coastal cliffs and islets in the breeding season, spending the wintertime in bays, estuaries, ports, or even in human buildings (SEO 2018). This species has experienced a dramatic increase in recent decades, due largely to its close link to human activities This can be easily observed along the coasts of islands, and they tend to concentrate in significant quantities in docks, dumps, and solid waste treatment plants (Martín and Lorenzo 2001). Their inhabiting patterns influence their feeding behaviors, which takes place mainly in coastal waters, but also in landfills and ports. Their diet includes predominantly fish, but also invertebrates, chickens and eggs from other birds, as well as carrion and rodents. As any other gull species, they can also feed on dumps and human remains (Watson 1981). Méndez et al. (2020) analyzed the diet of the yellow-legged gull chicks (*n* = 101) in Barcelona during the breeding season of 2018. These authors found that the main prey present in the stomach contents of the chicks consisted of urban birds, mainly rock pigeons and monk parakeets. According to IUCN (BirdLife International 2019, 2021) criteria, this species is considered a kind of “Least Concern” because of its wide distribution and the positive trend of its population (Figure S1). This species has an extremely large range, and hence does not approach the thresholds for vulnerable under the range size criterion. Indeed, mortality of individuals has been verified due to collision with power lines, consumption of toxic substances, etc. However, it is their population increase and predatory behavior that causes conservation problems to pelagic seabirds, especially those of small and medium size (Barone and Lorenzo 2007).

*Larus michahellis* are considered top predators and have a very wide feeding spectrum as they feed on disparate trophic chains and environments (Ramos et al. 2013). For years, studies evaluating pollutant levels have used accumulation levels in different tissues of seagulls (liver, kidney, blood, fat, bones), as good indicators of contamination levels. The most relevant tissues responsible for storage and detoxification of pollutants are liver and kidney (Lewis and Furness 1991). Birds can also rid their bodies of toxic substances through normal excretion and through deposition in the uropygial gland, salt gland, and in feathers (Burger and Gochfeld 1985, 1993; Braune and Gaskin 1987a, 1987b; Lewis and Furness 1991; Burger et al. 1992). On the other hand, feathers are capable of accumulating metals and organic pollutants from the atmosphere during their formation, giving information of a certain periodic exposure to contaminants (Burger et al. 1992, 2008; Burger 1993, 1996). Females can also sequester heavy metals in eggs and in eggshells (Fimreite 1974; Burger and Gochfeld 1993; Burger 1993; Gochfeld 1997).

Due to the ability of seabirds to travel long distances, their migratory behavior, and their establishment of fixed colonies in certain locations, these animals can serve as convenient indicators of environmental chemical pollution over large areas, in particular areas of inland and coastal waters. Some of the most relevant chemicals included in biomonitoring studies are metals. The appearance of metals in different tissues of animals often has been linked to specific urban areas (Korbecki et al. 2019). In fact, there is a need to carry out ecotoxicological studies to obtain data on specific species over a wider range. Recently, a significant elevation of metal concentrations in birds has been observed related to gross domestic products development. Specifically, the influence of the industrial development was reported as a critical reason for the increase in heavy metal levels in local avian species (Fu et al. 2014). These high concentrations of metals may lead to animal health impairment and a cumulative effect on the body with age, while also differences in tissue metal concentrations between sexes can occur (Barrales et al. 2021).

The main goal of the present study was to determine the validity of a gull species as a useful tool for biomonitoring metal levels in the environment. To reach this aim, we proceeded to assess the concentration of three heavy metals (mercury, cadmium, and lead) and two metalloids (selenium and arsenic) in the liver, kidney, and feathers of *L. michahellis* collected from three areas of Northwest Spain. These areas are located in Galicia and Asturias, with wide coastal and estuarine perimeters, two regions located along the Spanish coastline that are characterized by touristic, industrial, fishing, dredging and aquaculture activities, and/or suffering several contamination events in recent decades. They present well-preserved natural areas, but there are also some important cities and industrial plants (e.g., close to the cities of Pontevedra, A Coruña, Ferrol, and Gijón). Hg, Cd, and Pb were selected because they are the major contaminants of concern in marine environments (Fowler 1990). As, because of its concern for wildlife in marine and estuarine ecosystems (Neff 1997), and Se are known as a highly toxic element and an essential trace element, which bears a complex relationship with Hg, making it of high importance (Eisler 2000). Birds were collected during population control campaigns, duly authorized by the regional government of Galicia and Asturias, between 2013 and 2014, with no apparent signs or symptoms of injury or diseases or slaughtering in animal recovery centers. Different factors (tissue, sex, age, capture method, and sampling area) were considered during the data analysis to determine their relevance for future biomonitoring programs.

## Materials and methods

### Reagents

Solvents and reagents were of analytical grade or high purity grade and purchased from Panreac (Moncada i Reixac, Spain) and Merck (Darmstad, Germany). Milli-Q water was used to prepare solutions and dilutions. The internal standard used for elemental analysis by inductively coupled plasma mass spectrometer (ICP-MS) was a solution of yttrium, rhenium, rhodium, and tellurium (10 mg/L) purchased from Perkin Elmer Inc. (Shelton, USA).

### Sampling: study areas, species, and methodology

**Fig. 1:**
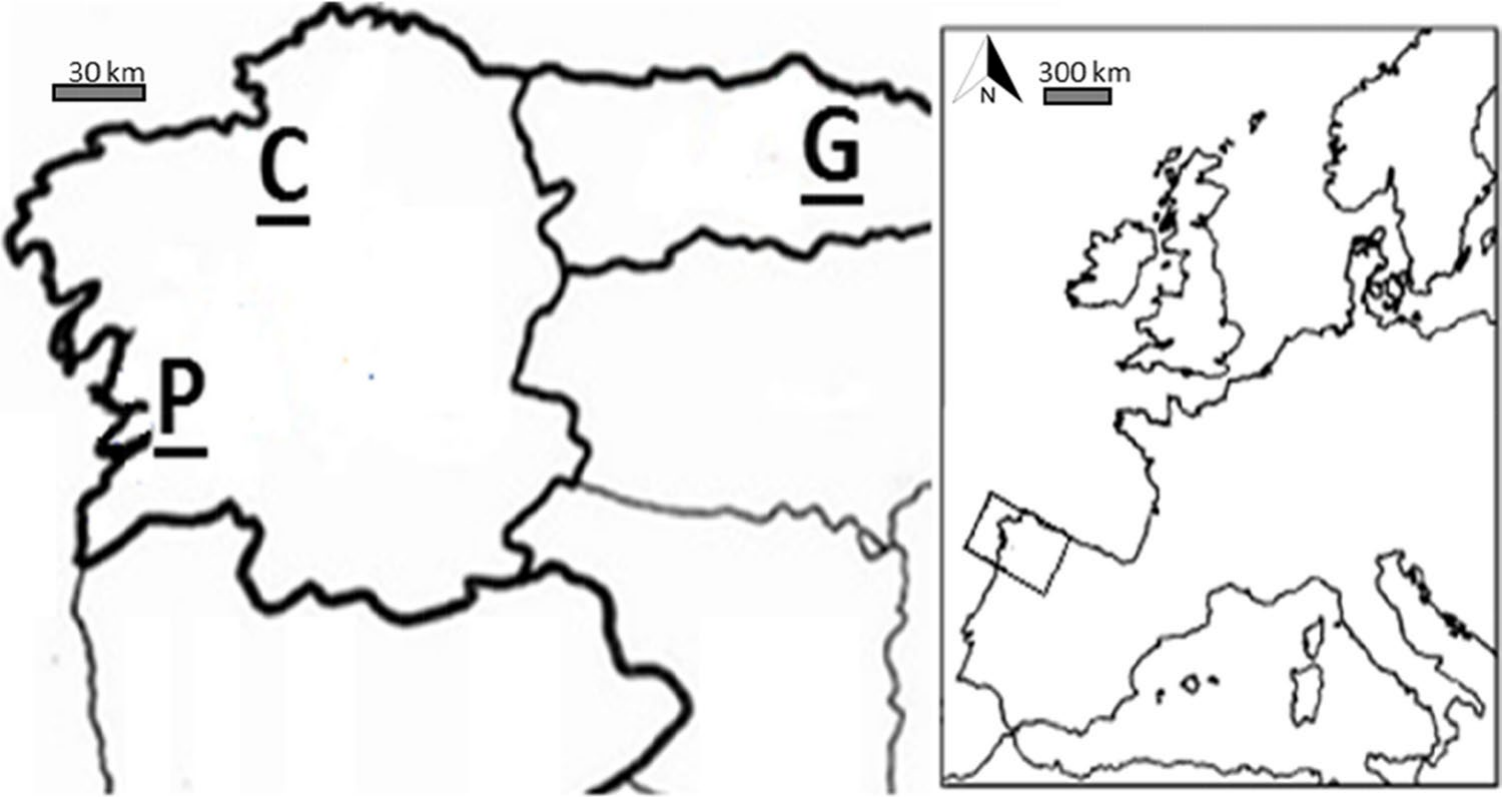
Geographical location: Pontevedra (P), A Coruña (C), and Gijón (G). NW Spain

The area of study was located in the northwest of Spain (Fig. 1), which was divided in three zones: Pontevedra (P), A Coruña (C), and Gijón (G). The distance from Pontevedra to A Coruña is 134 kms, and there is 282 km from A Coruña to Gijón.

Samples were collected from two different sources. One group of *L. michahellis* (*n* = 43) was collected in wildlife recovery centers from each area. Gulls were collected during the period of 2014–2016. Samples collected in the wildlife recovery centers were from birds that entered there mainly because of physical injuries, provoked by electrocution and fall from the nest due to inexperience in flying. Only birds held at the rehabilitation center for less than 5 days before dying were used (*n* = 43). The other group of seagulls (*n* = 66) came from population control campaigns in different populations, duly authorized by the regional governments of Galicia and Asturias, with no apparent signs or symptoms of injury or disease. During necropsy, several parameters such as body mass measurements, organ weights, bill development, and physical condition were registered. After adequate euthanasia (pentobarbital, 1 mL/kg, intravenous), the liver, kidney, and feathers (breast feathers) from each animal were collected and stored in individual zip-lock plastic bags, properly labeled and immediately frozen at -20 °C. Proper cleaning practices and replacing of surgical material were performed to avoid metal contamination. For feathers, the cleaning method consisted of washing with tap water, distilled water, Milli-Q water, and acetone.

Each animal was identified with information of interest (origin, cause of admission, age, sex, etc.). The animals were divided into 3 age groups: adults (*n* = 63), juveniles (*n* = 22), and chicks (*n* = 24) based on the color of plumage. Adult gulls have yellow legs, yellow beak with red spot on tip and yellow eye, their top feathers are grey, and the bottom ones are white. However, juvenile gulls have pink legs, dark beak, and eye of brown color, having plumage of brown color with dark striations. Chicks are attended by both adults for about 45 days, until they become independent and start to fly and are considered juveniles.

Samples were also grouped according to sex (55 males, 54 females). Both sexes are similar in plumage, although males have larger sizes compared to females. Indeed, males presented with wings greater than 465 mm, beaks greater than 62 mm, and tarsi greater than 75 mm. Females had wings shorter than 440 mm, beaks smaller than 54 mm, and tarsi with less than 64 mm. Visual checking of the gonads during the necropsy allowed to differentiate the samples by sex. The remains were hygienically removed by incineration, under current legislation.

Samples were sent to the Veterinary Faculty of Cáceres (Spain) in perfect condition, without breaking the cold chain.

### Determination of metals and metalloids

Samples of 3–4 g of liver and kidney were dried in an oven for 72 h at 65 °C. Feathers were washed with deionized water and NaOH 0.25 M to eliminate any possible contamination adhered to the surface. After this, the feathers were dried for 24 h at 40 °C. Finally, the feathers were lyophilized for 48 h and frozen at—80 °C for 24 h.

The metal levels were analyzed at the Elemental and Molecular Analysis Laboratory of the Research Support Service (SAIUEX, accredited by ISO 9001:2008) belonging to the University of Extremadura*.* A microwave assisted acid digestion procedure was carried out to obtain a suitable sample for metal content evaluation (adapted from Shah et al., 2009). Briefly, two grams of each sample were directly weighed into Teflon PTFE flasks and digested with 6 mL of a freshly prepared mixture of concentrated HNO_3_ (69%) and H_2_O_2_ (30%) (3:1, v/v) (supplied by Fluka) in a microwave digestion system and diluted to 10 mL with deionized water. The flasks were closed and kept for 10 min at room temperature. A blank sample was treated in the same way. All sample solutions were clear. The accuracy of the microwave digestion method was checked with a standard reference material (BCR® certified reference materials—ref. 185R, Community Bureau of Reference, EU). Following digestion, samples were transferred to 25 ml volumetric flasks. Digestion conditions for the microwave system are detailed in Table S1.

The analyses of liver and kidney samples were conducted by means of ICP-MS (Model 7900. Agilent Tech), following the operation conditions detailed in Table S2. This technique was selected because it provides sufficiently low detection limits and allows the simultaneous determination of several metals. For an optimal nebulization of the sample, a Peltier-cooled (2 °C) cyclonic chamber and a low-flow (0.25 mL/min) Meinhard Concentric Nebulizer were employed. Both the collision gas and the argon for the plasma have a purity of 99.999% and have been supplied by Praxair (Madrid, Spain).

Every working day, the ICP-MS was calibrated to obtain the highest values of intensity, indicated by the ratios CeO/Ce < 2.5%, Ce^2+^/Ce < 3%, and background (220) < 1 cps. Calibrating solutions were prepared daily from a 10 mg/L Multielement Calibration Standard 3 (PerkinElmer, Inc., Shelton, CT) and assayed as the samples. A NIST SRM 1577b bovine liver certified sample was used for quality control of the analytical procedure. Recoveries obtained were between 92% for Hg and 107% for Se, and coefficients of variation for replicate samples (*n* = 5) were lower than 6.5%. Metal levels were not adjusted for percent recovery. Limit of detection (LOD) and of quantification (LOQ) were determined according to the ICH-Q2 guideline on method validation (Guideline 2005), after analyzing repeated blanks with the same procedure used for the samples, determining the standard deviation. The final values of both parameters were calculated taking into account the dilution factor and the weight of the samples, being in all cases lower than 0.003 and 0.009 mg/kg for LOD and LOQ, respectively. The coefficients of variation for replicate samples (*n* = 5) were lower than 5.3%. Analytical blanks were included in all the run batches of samples.

### Statistical analysis

The statistical software Prism 5 version 5.03 for Windows (GraphPad software, Inc., CA) was used to analyze the data. Results were expressed as mean ± standard error of the mean (SEM), and the level for statistical significance was defined as *p* < 0.05.

Since data did not show a normal distribution and the variances were not homogeneous, the statistical analyses were performed using a non-parametric Mann Whitney *U*-test, to evaluate the differences related to both sex and age. Data were also compared by pairs applying a one way ANOVA, followed by a Tukey’s multiple comparison test, to check for differences between either sex or age related to the sampling area, thus obtaining information about the joint effect that two factors can have. Finally, a Spearman test was performed to determine the correlations among chemical levels. Statistical assessments were limited to those chemicals that could be detected in > 50% of the samples. For statistical testing, a value of 50% of the limit of detection (LOD) was assigned to samples with metal concentrations below LOD, thus minimizing nominal type I error rates (Clarke 1998).

## Results and discussion

### Concentration of metals/metalloids in bird’s tissues

The present work evaluated the levels of 3 metals and 2 metalloids in the liver, kidney, and feathers. Table 1 shows the results for the selected metals, being expressed as mg/kg dry weight (dw), since dry values are more reliable and consistent compared to wet weight (ww) values (Adrian and Stevens 1979). Data mentioned along the manuscript is related to dw, when data is mentioned as ww it is indicated.

**Table 1:** Concentrations of metals (Hg, Cd, Pb, Se, and As) in *L. michahellis* (mg/kg dw) (*N* = 109)

*Metal*	*Liver*		*Kidney*		*Feather*		*Total*
	*Mean* + *SEM*	*Median (range)*	*Mean* + *SEM*	*Median (range)*	*Mean* + *SEM*	*Median (range)*	*Mean* + *SEM*
***Hg***	2.95 ± 0.21	2.5 (0.34–16.39)	2.94 ± 0.18	2.60 (0.22–11.32)	1.13 ± 0.08	0.87 (0.18–5.92)	7.01 ± 0.37
***Cd***	4.13 ± 0.59	2.61 (0.11–50.9)	18.56 ± 2.46	9.25 (0.15–149.6)	0.12 ± 0.03	< LOD (< LOD–2.39)	22.82 ± 2.83
***Pb***	0.55 ± 0.77	0.41 (0.03–7.89)	2.50 ± 0.78	0.95 (0.7–79.81)	4.38 ± 1.09	1.61 (0.22–103.5)	7.36 ± 1.36
***Se***	7.18 ± 0.33	7.34 (0.31–15.91)	10.92 ± 0.42	10.95 (0.73–23.29)	0.54 ± 0.02	0.57 (< LOD–1.18)	18.64 ± 0.63
***As***	6.05 ± 0.39	5.34 (0.39–23.56)	4.54 ± 0.27	3.85 (0.45–13.93)	0.04 ± 0.01	< LOD (< LOD–1.46)	10.64 ± 0.59

The higher concentrations of Cd in kidney found in the yellow-legged gulls, compared to the liver, have been already shown in other avian species (Bianchi et al. 2008; Abdullah et al. 2015) and reported as a consequence of chronic exposure to low Cd concentrations (Scheuhammer 1987). Higher levels of metals in kidney than in liver were also observed for Se and Pb in the present study. There are not many studies assessing the Pb levels in gulls, but the collected data also revealed that in general Pb values are higher in the kidney compared to liver and feathers (Vizuete et al. 2019). The levels of Hg were similar in the liver and kidney. Arsenic levels were higher in the liver than in the kidney. In feathers, the levels of Pb were the highest of the analyzed elements. This is supported by other recent studies developed with seagulls, as showed by Agusa et al. (2005) in *L. crassirostris* from Japan, or by Mansouri et al. (2012) in *L. heuglini* from Iran.

A recent study developed with yellow-legged gull in the seabird colonies in the Atlantic Islands National Park and reported results of metals content in some biomaterials generated (feathers, fecal material, eggshells, etc.). The present median results of metals in feathers are higher than those reported by Otero et al. (2018), which can be explained by the higher pollution existing in our sampling areas. Moreover, these authors concluded that some biomaterials generated, like the excrement and pellets, contained high concentrations of trace elements. Thus, higher levels of metals may be found in the biomaterials generated by our animals, which will be deposited in their living areas.

Species located on the bottom part of the food chain should have lower tissue levels of heavy metals, because they accumulate less metals through ingestion. Indeed**, **Sarkka et al. (1978) found that Hg levels increased through the food chain, from water to plants and plankton, and then, to benthic predators. Several studies that have determined the levels of heavy metals in birds and their food have demonstrated that predatory birds have significantly higher levels than their prey, due to the magnification of these contaminants in the food chain (Lindberg and Odsjo 1983). Some studies are oriented to determine basal levels of metals, which provide useful data for biomonitoring purposes. In this sense, in wild birds living in environments with little or no industrial activities, the threshold reported for Hg levels in the liver was 10 mg/kg (Fimreite 1974). Even when some animals surpassed this threshold, our results (mean and median) of Hg in liver and kidney were below the mentioned threshold and were in accordance with those found by Leonzio et al. (1986) in *L. ridibundus* in Italy (2.58 ± 1.37 and 2.49 ± 1.71 mg/kg) and Majidi et al. (2015) in *L. heuglini* in Iran (1.87 ± 0.18 and 1.97 ± 0.21 mg/kg). However, these levels are lower than those found by Leonzio et al. (1986) in liver and kidney of *L. argentatus* in Italy (13.30 ± 9.25 mg/kg in liver; 10.65 ± 7.82 mg/kg in kidney), who suggested that the food source (marine areas) was responsible for the high Hg levels. The authors suggested that feeding patterns in dumps may explain the lower Hg levels in *L. ridibundus*, and this may explain the levels found in birds of the present study (Hg in feathers: 1.13 ± 0.08 mg/kg), which may feed in the human dumps instead of going to the coast. Factors other than food can also influence the Hg levels in feathers, like metabolic changes such as molting during which birds may eliminate a substantial part of Hg through their plumage, thus decreasing the internal tissues levels as they are sequestered in the feathers (Honda et al. 1986; Braune and Gaskin 1987a; Lewis and Furness 1991). Most of the animals were captured during the summer (August), months before the molt, since it is in October where these gulls eliminate the feathers. Nevertheless, our levels agree with those reported in the literature, with a range of Hg concentrations in feathers of seagulls between 0.29 ± 0.18 μg/g in *R. tridactyla* in Alaska (Burger et al. 2008) and 6.06 ± 4.60 μg/g in *L. argentatus* in Siberia (Kim et al. 1996), as showed in a recent review study (Vizuete et al. 2019).

Another metal evaluated was Cd, an extremely noxious pollutant that can be easily absorbed by seabirds from fish and marine invertebrates, and that accumulates in various tissues, including the kidney and to a lesser extent the liver and muscle (Kojadinovic et al. 2007). In the present study, Cd levels (4.13 ± 0.59 and 18.56 ± 2.46 mg/kg in liver and kidney, respectively) agree with those found by Leonzio et al. (1986) in *L. ridibundus* in Italy (4.20 ± 4.22 mg/kg in liver and 17.28 ± 9.92 mg/kg in kidney). However, 4 to 10 times higher levels in both organs have been reported in other gull species, like *L. ridibundus*, *L. crassirostris*, and *R. tridactyla* (Kim et al. 1996; Agusa et al. 2005; Braune and Scheuhammer 2008). The present Cd concentrations are also lower that those detected by Nielsen and Dietz (1989) in a population of adult Kittiwake considered healthy (76 mg/kg in the liver and 26 mg/kg in the kidney). The high levels of this metal described in gulls may be due to the diet of these animals, mainly consisting of squid, which represents an important source of cadmium for both seagulls and animals high up in the trophic chain (Bustamante et al. 1998). The process of detoxification and storage of non-essential elements in these animals could be confirmed by the high accumulation of Cd in the kidney (Lucia et al. 2008). Levels of Cd higher than 3 ppm in liver have been associated with health impairment and an increase in environmental contamination (Scheuhammer 1987; Burgat 1990). In the present study, the mean concentration of hepatic Cd was found to be slightly higher than this threshold (4.13 ± 0.59 mg/kg), suggesting a future adverse effect for the animal. In general, chronic exposure to low concentrations usually results in a high concentration of Cd at the renal level (Scheuhammer 1987). Some authors have used the kidney/liver ratio as an indicator for recent Cd exposure (Honda et al. 1986; Kim et al. 1996; Navarro et al. 2010). In the present study, the calculated kidney/liver Cd ratio was 4.31, which indicates a recent exposure to Cd. This result could be a good indicator of accumulation related to a specific area, since Cd resides for longer periods in kidney stores compared to liver stores (Honda et al. 1986; Kim et al. 1996; Navarro et al. 2010). These kidney/liver Cd ratios have also been reported by other authors, with levels between 1.5 in *L. ridibundus* in Poland (Orlowski et al. 2007) or 9.6 in *L. savini* in Siberia (Kim et al. 1996). The levels of Cd found in feathers were low (0.12 ± 0.03 mg/kg), something expected, since this tissue has been reported as less significant/a non-main-target for Cd accumulation (Frantz et al. 2016). Our levels are similar to those reported in two other studies developed in the Iberian Peninsula. Navarro et al. (2010) obtained 0.19 ± 0.002 mg/kg in *Phalacrocorax carbo* (Murcia, Spain), Otero et al. (2018) found 0.10 ± 0.07 mg/kg in *Larus michahellis* (Atlantic Islands of Galicia National Park, Spain), and Mendes et al. (2008) obtained 0.07 ± 0.02 mg/kg in feathers of *Morus bassanus* (Portugal)*.*

Poisoning by ingestion of objects that contain lead (Pb) is one of the most common causes of death in birds, especially seabirds. Lead pullets are one of the fastest and most important forms of contamination of this metal in the environment. It can be ingested by animals and moved through the trophic chain or can be disseminated in wetlands, producing pellet ingestion, since these bullets can confuse other animals due to their resemblance to seeds (Bellrose 1959; Sandersons and Bellrose 1986; Pain 1987).

In this study, levels of Pb measured were 0.55 ± 0.077 mg/kg and 2.50 ± 0.78 mg/kg, in the liver and kidney, respectively. These concentrations were also lower than those reported by Leonzio et al. (1986) in other species of seagulls (*L. ridibundus*), who found one of the highest lead concentrations in the kidney (30.96 ± 22.81 mg/kg) and liver (7.65 ± 5.41 mg/kg) recorded in seagulls of Europe. However, higher levels have been reported in birds found dead because of lead poisoning after ingestion of lead bullets (Perco et al. 1983). In general, the concentrations found in the present study for Pb in the liver and kidney can be considered of no toxicological relevance. Indeed, Pb concentrations ranging from 0.5 to 5.0 mg/kg in the liver and from 1.0 to 10.0 mg/kg in the kidney have been reported as background levels for seabirds from uncontaminated areas (Kehrig et al. 2015; Scheuhammer 1987). Agreeing with that, Pain et al. (1995) determined that Pb concentrations below 2 mg/kg in the liver could be safe and considered as a threshold value. Concentrations > 6 mg/kg were indicative of elevated exposure to lead in raptors capable of impairing biological functions, > 20 g/kg indicated lead poisoning, and concentration of > 30 g/kg in hepatic tissue which is considered a potentially lethal level (Pain et al. 1995). The levels of Pb in our samples of feathers were 4.38 ± 1.09 mg/kg, a high level in comparison to the 0.83 ± 0.37 mg/kg in the same species (Otero et al. 2018), or the 0.399 ± 0.048 mg/kg in *Morus bassanus* (Nardiello et al. 2019), both sampled in Galicia (Spain). Similar to that observed in the present study, the highest concentration of Pb was quantified in the feathers of *Morus bassanus* (Nardiello et al. 2019), in comparison with renal and hepatic levels. Pb concentrations of 4 mg/kg in the feathers are known to be the threshold levels of toxicity (Burger and Gochfeld 2000); thus, our recorded levels surpassed this threshold and, therefore, are of concern.

When Se is present as a pollutant in the environment, it can cause numerous harmful effects in birds. Mainly, these effects are mortality, impaired reproduction, and/or alterations in hepatic glutathione metabolism. In birds that survive the last phase of selenium poisoning, bleeding into the liver is very common. Se is a geogenic pollutant (i.e., it is naturally present in the rock and passes into the water during drilling carried out to collect water (for agricultural drainage waters)) (Hoffman 2002). Heinz (1996) suggested that concentrations > 3 mg/kg ww in the liver may impair reproduction, that sublethal toxic effects in birds can occur when the concentration of Se reaches 10 mg/kg ww (approximately 30 mg/kg dw), and finally that mortality could occur when hepatic concentration of Se reaches ≥ 20 mg/kg on a wet-weight basis (60 mg/kg dw). In the current study, the values of Se obtained in liver were 7.18 ± 0.33 mg/kg and 10.92 ± 0.42 mg/kg in kidney. These values are higher than those obtained by Dietz et al. (1996) in hepatic and renal tissue of *L. glaucoides* in Greenland, where an average concentration of 2.02 ± 1.4 mg/kg in the liver and 4.6 ± 1.49 mg/kg in the kidney were found. We also observed differences with respect to the study carried out on *R. tridactyla* species in Norway, with liver concentrations of 2.4 ± 9.8 mg/kg (Savinov et al. 2003), or the study carried out in Italy by Leonzio et al. (1986), in which mean concentrations at the renal level of 26.8 ± 9.92 mg/kg were detected in *L. argentatus*. These different values suggest the importance of this metalloid in body tissues depending on the geographical area. Indeed, Dietz et al. (1996) detected higher concentrations of Se in high trophic levels of different animals in Greenland, a relatively uncontaminated area compared with urban areas (Anderson et al. 2009).

Levels of Se in feathers are associated with toxic effects, ranging from 1.8 mg/kg (sublethal) to 26 mg/kg (lethality), depending upon the species (Burger 1993). Specifically, the levels of Se in feathers of *L. michahellis* have been reported from 3.0 to 5.8 mg/kg, which suggests potential adverse effects. Higher levels of Se have been reported in shorebird tissues, including feathers (Ohlendorf et al. 1989; Ackerman and Eagles-Smith 2009). However, the Se levels found in feathers in the present study (0.54 ± 0.02 mg/kg) are below the range reported as hazardous.

A potential protective effect has been reported for Se, which can reduce Cd-mediated toxicity in the liver and kidney in animal models and in cell culture studies. In this sense, Se antagonizes the toxicity of Cd mainly through sequestration of this element into biologically inert complexes and/or through the action of Se-dependent antioxidant enzymes (Zwolak 2020). The accumulation of Se found in the present study, therefore, could act as a protective element against the expected impairment generated by the Cd levels (above the hazardous threshold).

Inorganic As has been reported as highly toxic in some seabirds, by acting as an endocrine disruptor (Kunito et al. 2008). Arsenic concentrations are usually low (1 mg/kg ww, which approximately represents 3 mg/kg) in most living organisms (Braune and Noble 2009). Hepatic As values of 0.82 ± 0.26 mg/kg and 98.1 ± 69 mg/kg were found in *R. tridactyla*, in Norway and Russia, respectively (Savinov et al. 2003). In the present study, the concentration of As was 6.05 ± 0.39 mg/kg in the liver and 4.54 ± 0.27 mg/kg for the kidney. The concentrations of As in feathers was below the detection limit in almost all the samples, except for two individuals whose values were relatively low 1.46 and 0.15 mg/kg.

### Concentration of metals/metalloids according to sex

**Fig. 2:**
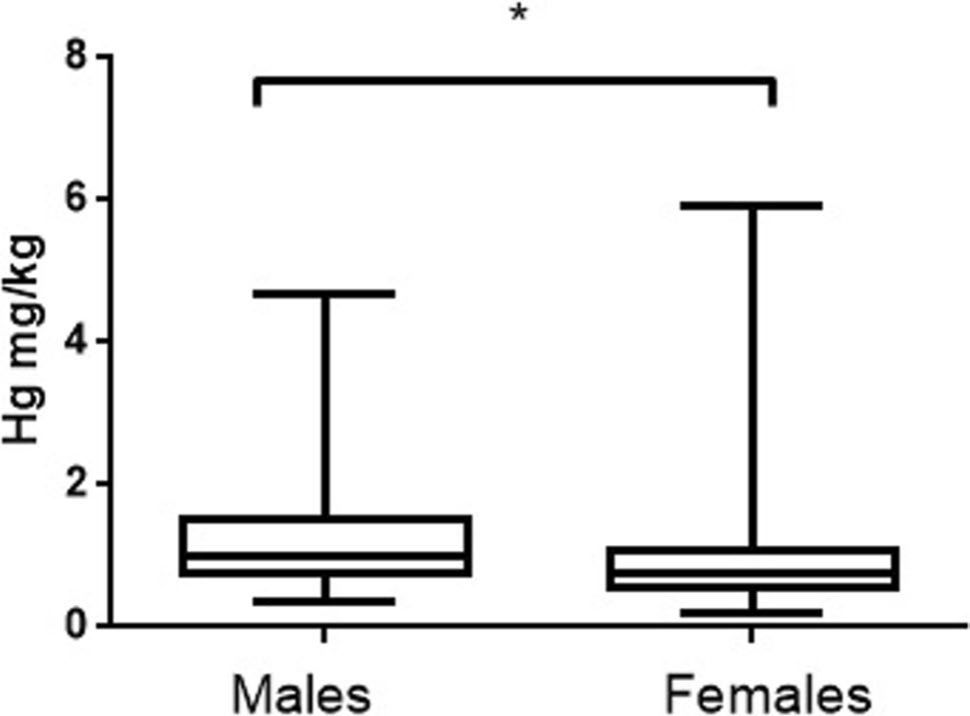
Hg median levels in feathers of yellow-legged gull according to sex (males, females). Box plots represent median values and 25 to 75% percentile ranges. *p < 0.05

Regarding the sex as an influencing factor, there were no significant differences in the liver and kidney concentrations of Hg, Cd, Pb, Se, and As between males and females. This was unexpected, since, for example, female birds should have lower concentrations of Hg than males, because breeding females can depurate methylmercury to their eggs (Ackerman et al. 2016). However, as already mentioned, the selected seagulls did not show sex differences in body burden of Hg, even if males tended to have higher levels of Hg both in liver and kidney. On the other hand, the levels of Hg in feathers were significantly higher (*p* < 0.05) in males than in females (Fig. 2). This fact was reported for Pb in a study of laughing gulls (*L. atricilla*) culled near a major airport, where males had barely significantly (*p* < 0.05) higher levels than females (Gochfeld et al. 1996), but there were not sex-related differences for the other heavy metals. Our levels of Pb were higher in males than in females in both organs, but we did not find significant differences. Only a few studies have suggested differences in levels related to sex, such as Hutton (1981) and Stock et al. (1989) in a study with oystercatchers; however, differences were found in opposite directions, making it difficult to make biologically meaningful conclusions.

A second factor, considered in the present study as a potential reason for different metal content, was the sampling area. The yellow-legged gull (*L. michahellis*) distributes in the circum-Mediterranean region, including the Macaronesian region. It has expanded along the Atlantic coast and to some humid areas of Central Europe (Geroudet 1984). These seagulls present an easy adaptation to their chosen habitat, which comprises of a variety of locations, such as marshes, beaches, and coastal islands. They live also in the vicinity of the coastal population centers, where they can successfully breed in human buildings. These gulls are not selective in terms of feeding, including in their diet fish, amphibians, mollusks, small mammals, and carrion. The two main feeding sources are the dumps and discard produced by fishing activity. There are several reasons for the implementation of culling plans to reduce their populations that include the following: their high reproductive success and ability to adapt to any environment, its pernicious effect on colonies, the negative effects on vegetation of the cliffs and potable water, noise problems, dirt, and damage to buildings (generated by urban colonies). Generally, this species is considered sedentary, as all of them remain close to their breeding colonies; however, some colonies go inland following the courses of the great rivers (SEO 2018).

### Concentration of metals/metalloid according to sampling area

**Fig. 3:**
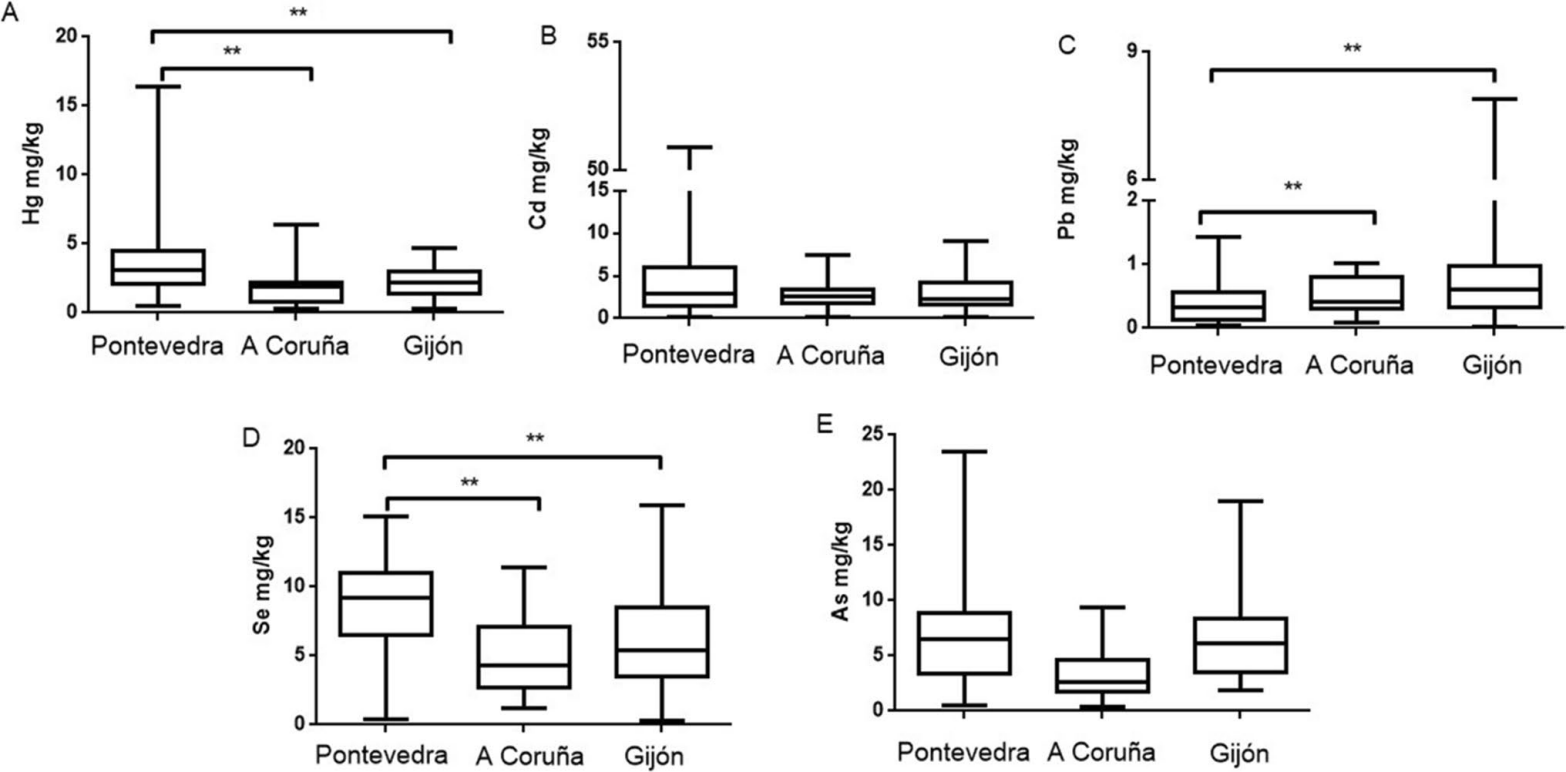
Hg (**A**), Cd (**B**), Pb (**C**), Se (**D**), and As (**E**) levels in liver of yellow-legged gull according to the three different sampling areas: Pontevedra, A Coruña, and Gijón. Box plots represent median values and 25 to 75% percentile ranges. **p < 0.01

**Fig. 4:**
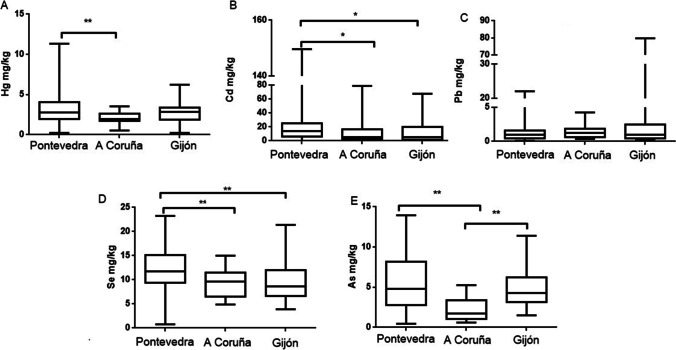
Hg (**A**), Cd (**B**), Pb (**C**), Se (**D**), and As (**E**) levels in kidney of yellow-legged gull according to the three different sampling areas: Pontevedra, A Coruña, and Gijón. Box plots represent median values and 25 to 75% percentile ranges.*p < 0.05, **p < 0.01

Significant differences in liver the (Fig. 3) and kidney (Fig. 4) Hg levels were found among sampling areas. In the liver, there are significant differences between Pontevedra and A Coruña and between Pontevedra and Gijón. Something similar occurs in the kidney, which presents also significant differences between Pontevedra and A Coruña. Hg pollution studies in animals are scarce in Galicia. Beiras et al. (2002) found different Hg concentrations in different areas of Galicia: 73.5 ng/L in seawater, 0.186 mg/kg in sediments, and 0.741 mg/kg in mussels in the Pontevedra Rias. These authors compared Hg concentrations in different coastal areas of Galicia, finding higher metal levels in the Rias of Pontevedra than in those of Vigo, A Coruña and Arousa. The present study also found higher levels in Pontevedra than in the other 2 regions. Galician wild mussels showed Hg contents of 0.05–0.2 mg/kg (Besada et al. 1997). Both studies demonstrate the persistence of local Hg pollution, despite recent efforts undertaken by the chlor-alkali industry aimed at reducing the levels of Hg in their effluent. The levels of Hg found in our gulls (Table 2) in livers were 3.71 ± 0.33 mg/kg, 1.79 ± 0.29 mg/kg, and 2.24 ± 0.21 mg/kg in Pontevedra, A Coruña, and Gijón, respectively. The same pattern was observed in the kidney (3.38 ± 0.31 mg/kg, 2.03 ± 0.18 mg/kg, and 2.72 ± 0.25 mg/kg). Hg enters the marine ecosystems through fluvial and atmospheric pathways both leading to the water column where, either dissolved or associated with small particles, it is available for suspension feeders such as marine mussels (Beiras et al. 2002), located in the low zone of the food chain. Regarding Cd levels, there were no significant differences in levels in the liver between the three studied areas; however, differences appeared in kidney tissues between Pontevedra and Gijón and between Pontevedra and A Coruña. These differences may be due to the fact that Cd mainly accumulates in the kidney and differences can be more marked when accumulated levels are higher. In both cases, Cd levels were higher in Pontevedra than in the other two areas. The higher levels of Cd in Pontevedra compared to the other areas may be due to inadequate waste water treatment, which fails to comply with current regulations on wastewater treatment. Even sediments and sludge produced in estuaries containing Cd as the main heavy metal makes Pontevedra one of the most polluted areas. The Court of Justice of the European Union ratified the repeated non-compliance with the treatment of wastewater in the Ria de Vigo (Galicia), where a poor treatment in its sanitation is being caused (https://ec.europa.eu/commission/presscorner; 17 November 2016).

**Table 2:** Metal concentrations in *L. michahellis* (mean ± SEM; mg/kg dw) according to sampling area, age and capture method

*Metal*	Metal concentration in *Larus michahellis* (mean ± SEM; mg/kg) according to							
	*Sampling area*			*Age*			*Capture method*	
	Pontevedra (*n* = 58)	A Coruña (*n* = 21)	Gijón (*n* = 30)	Adults (*n* = 63)	Juveniles (*n* = 22)	Chicks (*n* = 24)	Control (*n* = 66)	Recovery center (*n* = 43)
	*Liver*			*Liver*			*Liver*	
*Hg*	3.71 ± 0.33	1.79 ± 0.29	2.24 ± 0.21	3.33 ± 0.22	3.13 ± 0.71	1.76 ± 0.28	2.36 ± 0.19	3.85 ± 0.41
*Cd*	5.11 ± 1.06	2.83 ± 0.38	3.14 ± 0.41	4.74 ± 0.62	4.97 ± 2.27	1.79 ± 0.2	4.32 ± 0.78	3.85 ± 0.90
*Pb*	0.38 ± 0.4	0.51 ± 0.06	0.88 ± 0.24	0.65 ± 0.12	0.4 ± 0.11	0.42 ± 0.06	0.64 ± 0.12	0.41 ± 0.05
*Se*	8.60 ± 0.39	4.97 ± 0.6	5.86 ± 0.62	7.56 ± 0.43	8.21 ± 0.72	5.24 ± 0.53	6.29 ± 0.41	8.56 ± 0.46
*As*	6.71 ± 0.60	3.40 ± 0.51	6.48 ± 0.62	5.81 ± 0.48	8.25 ± 1.21	4.67 ± 0.43	5.82 ± 0.51	6.41 ± 0.63
	*Kidney*			*Kidney*			*Kidney*	
*Hg*	3.38 ± 0.31	2.03 ± 0.18	2.72 ± 0.25	3.14 ± 0.22	3.1 ± 0.61	2.29 ± 0.25	2.65 ± 0.18	3.93 ± 0.37
*Cd*	23.63 ± 3.98	11.86 ± 3.91	13.39 ± 3.32	27.81 ± 3.77	9.07 ± 2.2	2.98 ± 0.46	16.71 ± 2.37	21.41 ± 5.09
*Pb*	1.58 ± 0.39	1.48 ± 0.26	4.86 ± 2.62	3.50 ± 1.35	1 ± 0.29	1.24 ± 0.22	3.04 ± 1.24	1.67 ± 0.51
*Se*	12.24 ± 0.61	9.34 ± 0.68	9.47 ± 0.69	11.75 ± 0.55	10.29 ± 1.24	9.13 ± 0.7	10.24 ± 0.46	11.97 ± 5.91
*As*	5.30 ± 0.41	2.85 ± 0.29	4.72 ± 0.4	4.77 ± 0.37	4.89 ± 0.69	3.65 ± 0.4	3.66 ± 0.30	5.91 ± 0.44
	*Feather*			*Feather*			*Feather*	
*Hg*	0.83 ± 0.06	0.98 ± 0.11	1.8 ± 0.23	0.87 ± 0.11	0.87 ± 0.11	0.87 ± 0.05	1.41 ± 0.12	0.69 ± 0.05
*Cd*	0.09 ± 0.03	0.026 ± 0.01	0.22 ± 0.07	0.15 ± 0.05	0.09 ± 0.03	0.07 ± 0.02	0.17 ± 0.04	0.03 ± 0.01
*Pb*	2.67 ± 0.46	1.88 ± 0.36	9.27 ± 3.68	4.24 ± 1.65	3.22 ± 0.82	5.83 ± 2.28	6.10 ± 1.76	1.71 ± 0.23
*Se*	0.42 ± 0.02	0.63 ± 0.05	0.69 ± 0.03	0.43 ± 0.04	0.43 ± 0.04	0.68 ± 0.03	0.60 ± 0.02	0.44 ± 0.03
*As*	0.04 ± 0.02	0.02 ± 0.01	0.05 ± 0.01	0.035 ± 0.01	0.03 ± 0.01	0.02 ± 0.01	0.05 ± 0.02	0.01 ± 0.01

Seagulls from Gijón showed higher levels of Pb, in both organs, compared to the other two regions. However, only significant differences were found in livers between Pontevedra and the other two areas. A factory that uses Pb for the manufacturing of acer is located in Gijón, being a potential source of this metal and a potential explanation for the higher levels of Pb in this area.

Significant differences can be observed in levels of Se between Pontevedra and A Coruña or Gijón, both in liver and kidney tissues. In both organs, Se levels were higher in Pontevedra. Ohlendorf et al. (1988) describes that the ratio liver/kidney for Se is about 1 for freshwater aquatic birds in Se-contaminated areas; our ratios did not reach this value, approaching levels of 0.70, 0.53, and 0.62 in Pontevedra, A Coruña, and Gijón, respectively (from Table 3). Therefore, it was considered that these areas did not present a high Se pollution. Finally, As levels show that gulls from Pontevedra accumulated more As in both organs, showing significant differences in A Coruña when compared to the other two areas.

**Table 3:** Correlations between the pairs of the studied elements into the specific organ (liver, kidney, and feathers) and between tissues for a specific metal

	*p* < *0.05*	*p* < *0.01*	*p* < *0.001*
*Liver*	*Cd-Se (r* = *0.23)*		*Hg-Cd (r* = *0.38)*
			*Hg-Se (r* = *0.36)*
*Kidney*	*Hg-As (r* = *0.20)*		*Hg-Cd (r* = *0.39)*
			*Hg-Se (r* = *0.33)*
			*Cd-Se (r* = *0.44)*
*Feather*	*Hg-As (r* = *0.23)*	*Hg-Cd (r* = *0.31)*	*Hg-Se (r* = *0.50)*
	*Cd-As (r* = *0.24)*	*Hg-Pb (r* = *0.26)*	*Cd–Pb (r* = *0.51)*
	*Pb-Se (r* = *0.24)*	*Pb-As (r* = *0.25)*	*Se-As (r* = *0.40)*
*Liver-kidney*			*Hg (r* = *0.46)*
			*Cd (r* = *0.68)*
			*Pb (r* = *0.46)*
			*Se (r* = *0.43)*
			*As (r* = *0.53)*
*Liver-feather*			*Pb (r* = *0.34)*

**Fig. 5:**
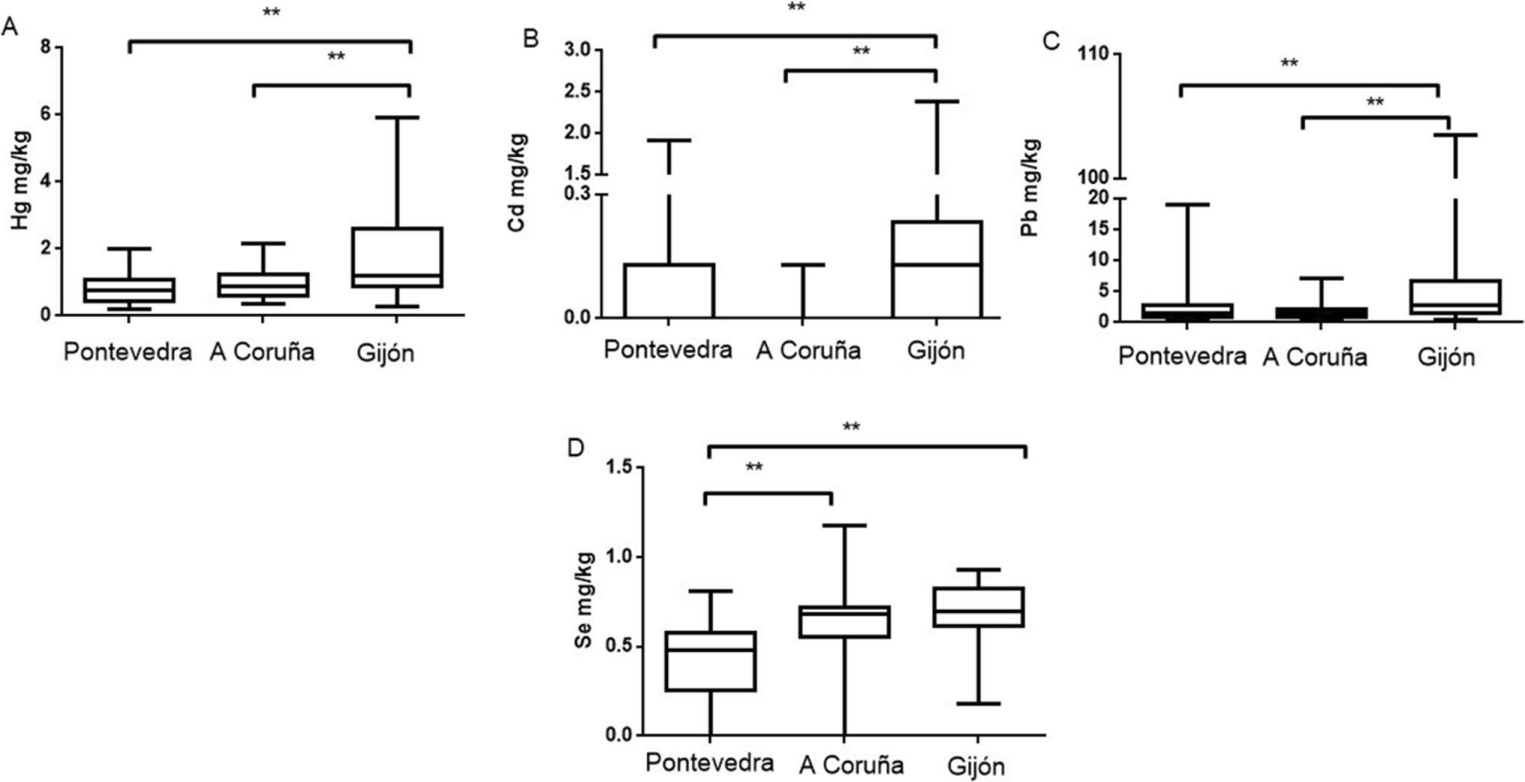
Hg (**A**), Cd (**B**) Pb (**C**), and Se (**D**) levels in feathers of yellow-legged gull according to the three different sampling areas: Pontevedra, A Coruña, and Gijón. Box plots represent median values and 25 to 75% percentile ranges. **p < 0.01. As levels could not be represented because many samples showed levels below the limit of detection

In general, feathers were the most consistent tissue to differentiate polluted areas. Seagulls from Gijon presented always higher levels of Hg, Cd, Pb, and Se than the other two sampling areas (Fig. 5). Korbecki et al. (2019) also reported the differences in metal levels (Pb) found in feathers depending on the studied area and species. In general, feathers have been reported as a potential non-invasive tool for metal biomonitoring in seabirds (Hernández-Moreno et al. 2021).

### Concentration of metals/metalloid according to age

**Fig. 6:**
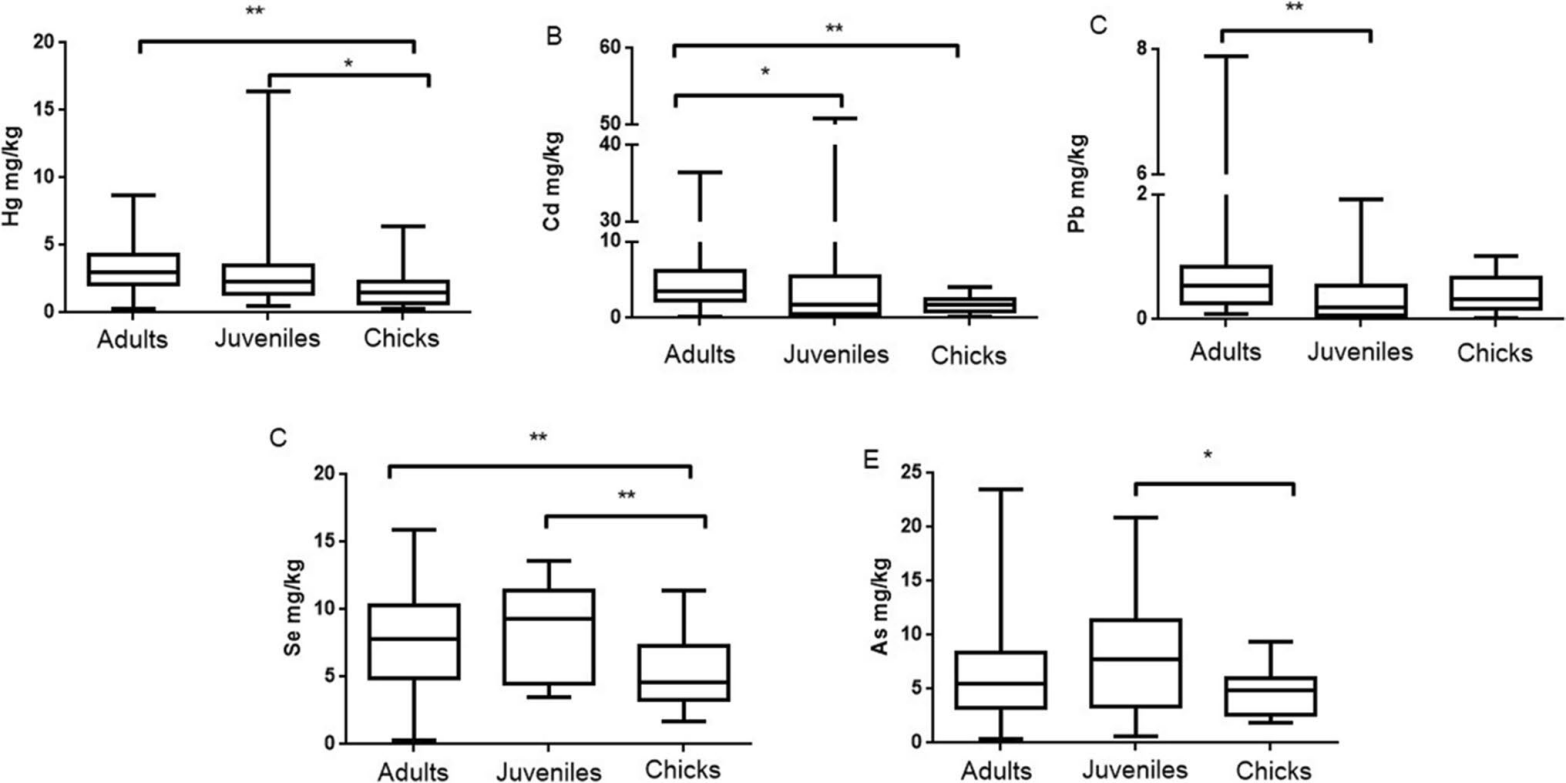
Hg (**A**), Cd (**B**), Pb (**C**), Se (**D**), and As (**E**) levels in liver of yellow-legged gull according to age: adults, juveniles, and chicks. Box plots represent median values and 25 to 75% percentile ranges. *p < 0.05, **p < 0.01

**Fig. 7:**
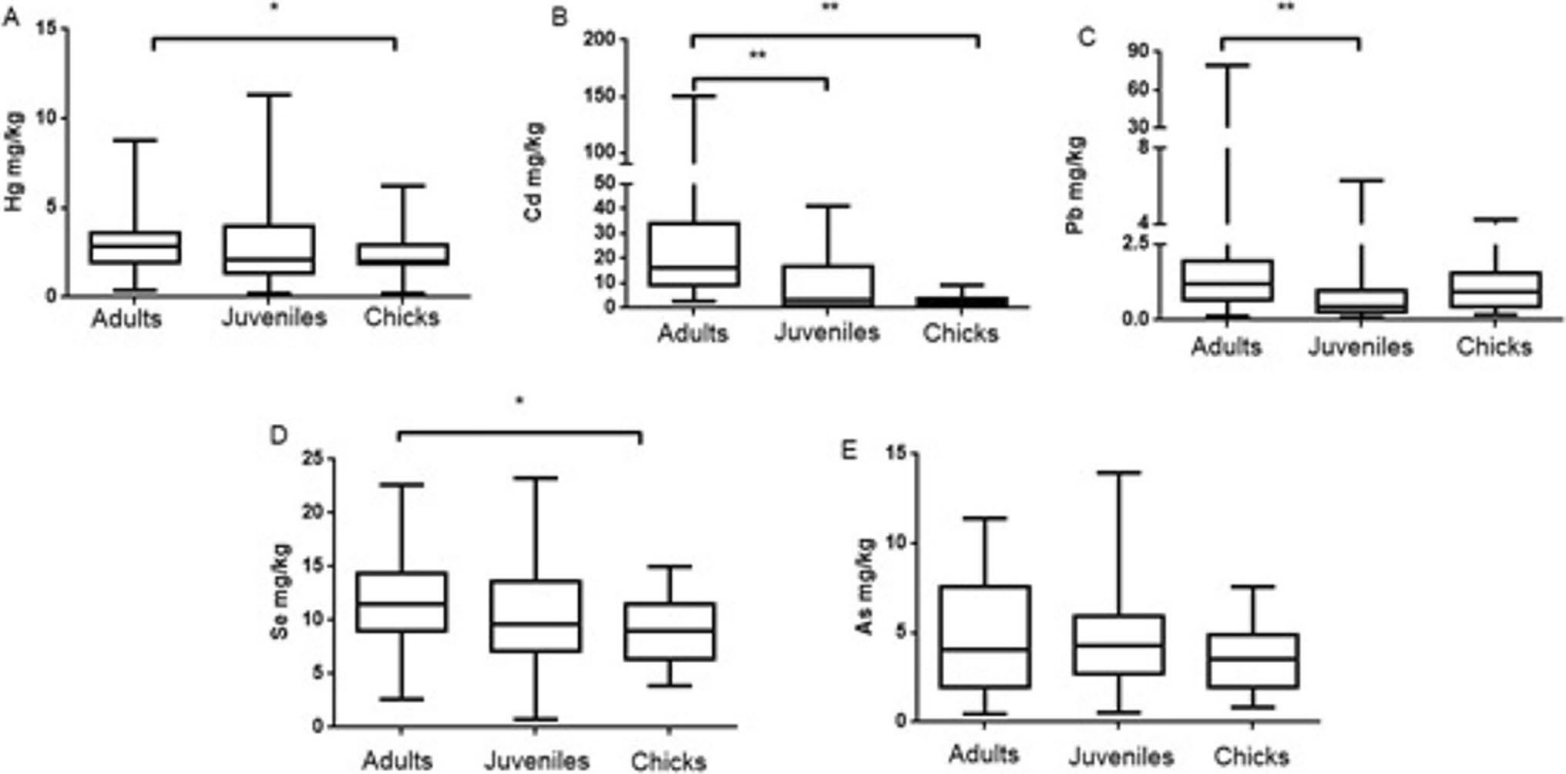
Hg (**A**), Cd (**B**), Pb (**C**), Se (**D**), and As (**E**) levels in kidney of yellow-legged gull according to age: adults, juveniles, and chicks. Box plots represent median values and 25 to 75% percentile ranges. *p < 0.05, **p < 0.01

**Fig. 8:**
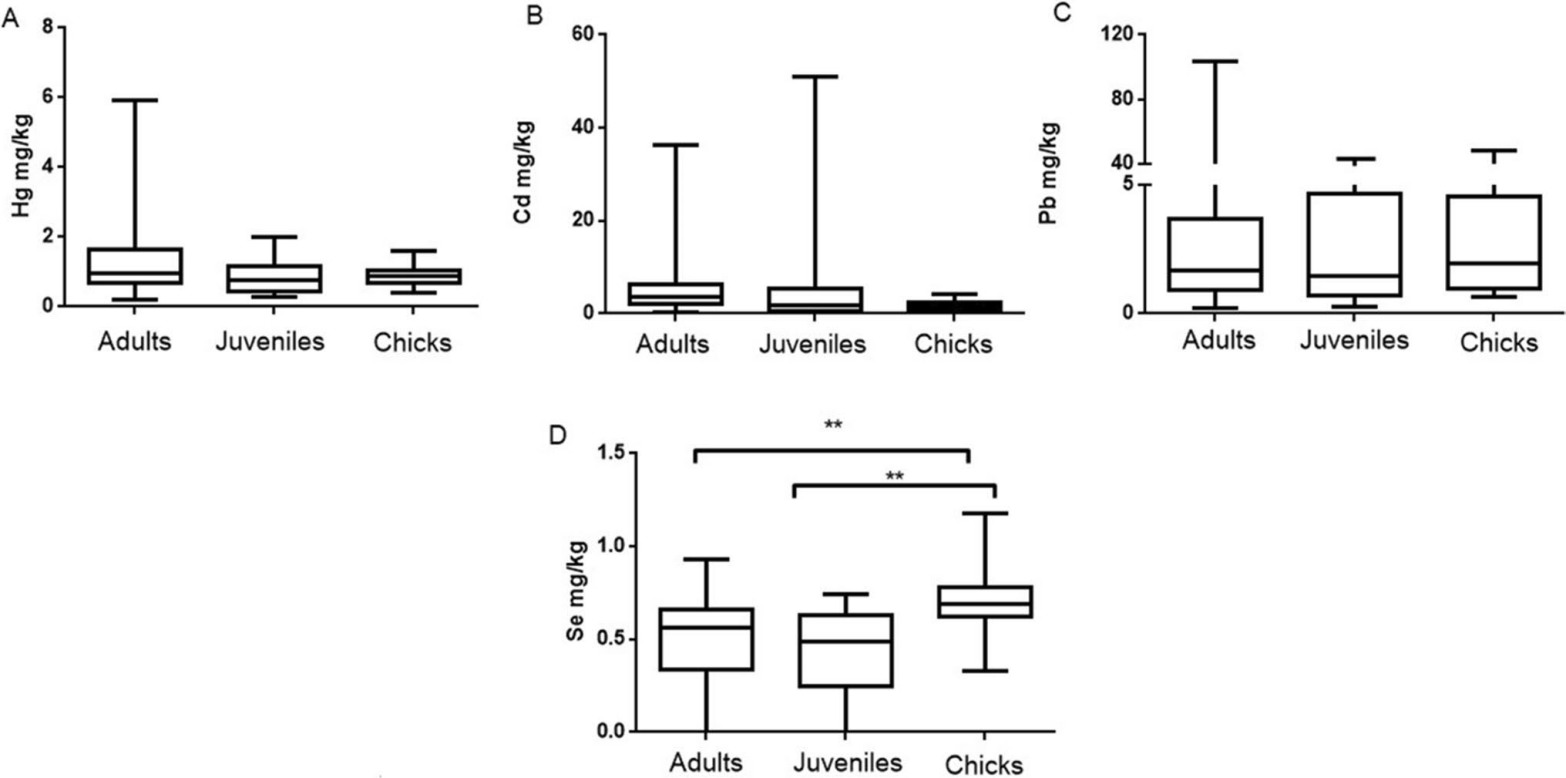
Hg (**A**), Cd (**B**), Pb (**C**), and Se (**D**) levels in feathers of yellow-legged gull according to age: Adults, Juveniles and Chicks. Box plots represent median values and 25 to 75% percentile ranges. **p < 0.01. As levels could not be represented because many samples showed levels below the limit of detection

The age as factor in the metal(oids) content in the liver, kidney, and feathers is shown in Figs. 6, 7, and 8 and Table 2. With increasing age, adult gulls are exposed to metals through food and water. Once ingested, contaminants can be directly excreted or absorbed into the circulatory system. Age-related differences were expected for most of the studied metals because adults had a longer life-time to accumulate them. This usually occurs in the case of Hg, Cd, and Pb in the liver. However, this increase was clearer in kidney tissues, where levels of Hg, Cd, Pb, and Se were significantly higher in adults than in juveniles and chicks. In agreement with the present results, Friberg et al. (1974) obtained age-related increased concentrations of Cd in several tissues of oystercatcher and great skua. An explanation for this accumulation related to age is the long biological half-time (15–30 years) of the metal, which is thought to be a consequence of the binding of Cd to metallothionein and its subsequent retention in the kidney (Friberg et al. 1974; Kowal et al. 1979). In addition, it is possible to observe greater age-related differences in the kidney than in the liver, mainly because the kidney is the target organ for Cd. On the other hand, Stewart et al. (1994) demonstrated seasonal changes in Cd levels in internal tissues of guillemots and highlighted some kind of regulation mechanism for this metal. Through regulation, Cd would not continue to accumulate with increasing adult age, and it would not result in older birds having higher Cd burdens than younger adult birds. Studies developed during the 1980s showed no age-related Cd accumulation in herring gulls (*L. argentatus*) (Hutton 1981; Nicholson 1981). However, more recent studies (Kim and Oh 2017) reported that adults of *L. crassirostris* presented higher levels of Cd than nestlings. Some other authors reported also higher liver concentrations of Cd in adult birds than in nestlings or juveniles for black-headed gulls (*L. ridibundus*) (Orlowski et al. 2007) and glaucous gulls (*L. hyperboreus*) (Migula et al. 2000).

Regarding Pb, levels were higher in adults, followed by chicks (without significance) and, lastly, the juveniles. The higher values found in adults may be due to the accumulation of this metal associated with age and their wide mobility to find food. The high levels found in chicks could be related to those in adults by direct transmission, once all of them are in the nest. Finally, the lower levels in juveniles may be explained by the metal elimination together with the feathers during molting. Pb concentrations are increased in those areas with high population, showing the importance of establishing monitoring programs in areas with a high density of buildings. Stripping paint off buildings without proper containment control of chips appears to increase the availability of Pb to chicks and compounds the problem (Work and Smith 1996).

Moreover, the juveniles of the present study have higher levels of Se and As in liver than the other two groups. The diet of these animals may explain the differences found between adults and fledglings. Changes in the diet of this species can take place during the breeding period, leading to a different nutrition between adults and young. In fact, the main diet of seagulls consist of invertebrates rather than fish, especially in chicks. However, when breeding period occurs, the adult gulls base their diet mainly on larger fish (Burger, unpublished data). On the other hand, Se and As show the normal pattern in the kidney, with higher levels in adults compared to juveniles and chicks.

In feathers, statistically significant differences for Se were found between chicks and adults or juveniles, with in both cases higher levels of Se found in chicks (Fig. 8).

### Concentration of metals/metalloid according to capture method

The last factor considered in this study was the influence of the capture method. Results showed different values depending on the tissue (Table 2). In the liver, Hg, Se, and As presented lower levels in birds captured during control campaigns than birds which died in recovery centers, with significant differences in Hg and Se levels. In the kidney, the levels of Hg, Cd, Se, and As were higher in animals from recovery centers in (levels of As were significantly different). However, the metal content in feathers was always higher in the animals captured in control campaigns, finding statistical differences (*p* < 0.001) for Hg, Cd, Pb, and Se.

When the comparisons were performed classifying samples by sex or age related to the specific location, some differences were found by pairs. By sex, differences were found mostly in feather content of Hg, Cd, Pb, and Se. In these cases, females from A Coruña (Hg (*p* < 0.05), Cd (*p* < 0.05 and 0.01), and Pb (*p* < 0.0001)) and Gijón (Hg (*p* < 0.05)) showed higher values than females and males from other areas. Regarding Se, males from Gijón and A Coruña presented the lowest values in feathers (*p* < 0.0001), whereas males from Pontevedra showed higher values of this metal in liver (*p* < 0.01). According to age, adults from Asturias showed higher values (*p* < 0.0001) of Hg in feathers than adults from Pontevedra, as well as from juveniles and chickens. For Cd, there was a clear higher value found in kidney from chickens of Asturias when compared to the other groups (*p* < 0.01 and 0.05). In A Coruña, levels of Pb were significantly higher in feathers of adult animals respect to the other ages and areas (*p* < 0.0001). Regarding Se, the previously reported lower values in feathers of juveniles in comparison to adults and chickens was confirmed, but no clear differences were found related to the sampling area.

### Correlations study

Regarding the correlations study, weak to moderate positive correlations were found between pairs of various studied metals (*r* = 0.3–0.5), as shown in Table 3. The liver and kidney showed correlations between Cd-Se, Hg-Cd, and Hg-Se, with the latter found also in feathers. The Hg-Se correlation in the three tissues might indicate a possible implication of the Se in the detoxification of metals, like Hg (Blanco et al. 2003). An analogous relationship between Hg and Se has not been as clearly demonstrated in marine birds, although there are many toxicity studies providing evidence of such a mechanism in the significant correlation of Se–Hg levels (Marier and Jaworski 1983). Many authors suggest that the protective effect of selenite on the toxicity of Hg^2+^ is mainly due to the formation of mercuric selenide (HgSe). This represents a stable and biologically inert complex (Koeman et al. 1973; Das et al. 2003; Eagles-Smith et al. 2008; Yang et al. 2008) that produces a decrease in the toxicity of Hg. A significant positive correlation between hepatic Hg and Se in gulls has been reported (Hutton 1981). Moreover, it has been suggested that both metals (Se and Hg) ameliorate the toxicity of the other (Eisler 2000).

Regarding other elements, the biological significance of the correlations between Cd and Se in seabirds is unclear. Pařízek et al. (1971) stated that this association may suggest a protective influence in the organisms due to the potential antioxidant activity of Se. In the present study, this correlation was found in liver and kidney.

However, no correlations were found between Pb and Se either in the liver or in kidney, showing a weak correlation in feathers (*r* < 0.3). The low levels of Pb may explain the lack of relationship, since Pb levels may not be high enough to increase the levels of metal-binding proteins such as protoporphyrins and metallothioneins (Elliott et al. 1992; Stewart et al. 1996). Nevertheless, a moderate to strong correlation was found in feathers between Pb and Cd (*r* > 0.5). This correlation has been already reported, as well as the lack of correlation of Hg with these two metals in feathers. The authors pointed out as a possible explanation the same origin of Cd and Pb, being different from that of Hg (Bianchi et al. 2008). In the present study, it was possible to establish a correlation between these metals in feathers (Hg-Cd and Hg-Pb) but with a weak r value.

Finally, strong positive correlations between the liver and kidney in levels of Hg, Pb, Se (weak to moderate), Cd, and As (moderate to strong) were found, as tissues representing major reservoirs and metabolization organs. There was also a positive correlation found between liver and feathers, pointing to a possible release of metal through this external tissue.

## Conclusions

In the present study, the results from accumulation assessment of Hg, Cd, Pb, Se, and As in the liver, kidney, and feathers of *L. michahellis* from 3 different areas (Pontevedra, A Coruña, and Gijón) provide evidence that the three tissues are adequate samples to understand heavy metals pollution levels and bioaccumulation along the wetland food chain. Metal concentrations detected during the present study were within or below the range of levels found for other seagulls. Although internalized metal levels will not provoke lethality to the selected animals, their identification in tissues denotes exposure through the environment. Indeed, it was confirmed that the liver, kidney, and feathers of *L. michahelli*s can reveal local contamination around the foraging and breeding sites. This makes the selected species of high interest as an instrument for assessing heavy metal contamination not only following a disaster but also for routine monitoring studies, both in coastal and inland areas. Therefore, *L. michahellis* can be a useful sentinel species of environmental health, providing an early warning of possible exposure for humans inhabiting the same ecosystems and often eating the same foods.

## Supplementary Information

Below is the link to the electronic supplementary material.

Supplementary file1 (DOCX 686 KB)

## Data Availability

The datasets used and/or analyzed during the current study are available from the corresponding author on reasonable request.

## References

[CR1] Abdullah M, Fascola M, Muhammad A, Ahmad BN, Bokhari H, Aqeel M, Nawz M, Alamdar A, Khan M, Ali N, Eqani S (2015). Avian feathers as a non-destructive biomonitoring tool of trace metals signatures: a case study from severely contaminated areas. Chemosphere.

[CR2] Ackerman JT, Eagles-Smith CA (2009). Selenium bioaccumulation and body condition in shorebirds and terns breeding in San Francisco Bay, California, USA. Environ Toxicol Chem.

[CR3] Ackerman JT, Eagles-Smith CA, Herzog MP, Hartman CA (2016). Maternal transfer of contaminants in birds: mercury and selenium concentrations in parents and their eggs. Environ Pollut.

[CR4] Adrian WJ, Stevens ML (1979). Wet versus dry weights for heavy metal toxicity determinations in duck liver. J Wildlife Diseases.

[CR5] Agusa T, Matsumoto T, Ikemoto T, Anan Y, Kubota R, Yasunaga G, Shibata Y (2005). Body distribution of trace elements in black-tailed gulls from Rishiri Island, Japan: age-dependent accumulation and transfer to feathers and eggs. Environ Toxicol Chem.

[CR6] Anderson O, Phillips R, McDonald R, Shore R, McGill R, Bearhop S (2009). Influence of trophic position and foraging range on mercury levels within a seabird community. Mar Ecol Progress Series.

[CR7] Arizaga J, Galarza A, Herrero A, Hidalgo J, Aldalur A (2009). Distribución y tamaño de la población de la Gaviota Patiamarilla *Larus michahellis* lusitanius en el País Vasco: tres décadas de estudio. Rev Catalana D'ornitologia.

[CR8] Baker S, Herrchen M, Hund-Rinke K, Klein W, Kordel W, Peijnenburg W, Rensing C (2003). Underlying issues including approaches and information needs in risk assessment. Ecotoxicol Environ Saf.

[CR9] Barone R, Lorenzo JA (2007). Gaviota patiamarilla, *Larus michahellis*. Dirección General de Conservación de la Naturaleza - SEO/BirdLife, Editors: Juan A. Lorenzo, pp 245–249.

[CR10] Barrales I, Hernández-Moreno D, Fidalgo LE, López-Beceiro A, Martínez-Morcillo S, Sánchez-Montero L, Míguez MP, Soler F, Pérez-López M (2021). Levels of zinc, cadmium, and lead in liver, kidney, and feathers of Atlantic puffins (*Fratercula arctica*) from Spain. Toxicol Environ Chem.

[CR11] Beiras R, Fernández N, González JJ, Besada V, Schulte F (2002). Mercury concentrations in seawaters and wild mussels from the coast of Galicia (NW Spain). Mar Pollut Bull.

[CR12] Bellrose FC (1959). Lead poisoning as a mortality factor in waterfowl populations. Ill Nat Hist Surv Bull.

[CR13] Besada MV, Fumega J, Cambeiro B (1997). Variación anual delas concentraciones de Hg, Pb, Cd, Cu y Zn *en mejillon silvestre de la Ria de Vigo*. In: Prego, R., Fernández, J.M. (Eds.),* Procesos biogeoquimicos en* sistemas costeros Hispano-Lusos. Diputación Provincial de Pontevedra, Pontevedra, pp. 95–99, ISBN.: 84–89690–27–8.

[CR14] Bianchi N, Ancora S, Difazio N, Leonzio C (2008). Cadmium, lead and mercury levels in feathers of small passerine birds: non invasive sampling strategy. Environ Toxicol Chem.

[CR15] BirdLife International (2019) Species factsheet: *Larus michahellis*. Downloaded from http://www.birdlife.org on 27/05/2019

[CR16] BirdLife International (2021) Species factsheet: Larus michahellis. Downloaded from http://www.birdlife.org on 14/07/2021

[CR17] Blanco G, Frias O, Jimenez B, Gomez G (2003). Factors influencing variability and potential uptake routes of heavy metals in black kites exposed to emissions from a solid-waste incinerator. Environ Toxicol Chem.

[CR18] Braune BM, Gaskin DE (1987). Mercury levels in Bonaparte's Gull (*Larus philadelphia*) during autumn molt in the Quoddy region, New Brunswick, Canada. Arch Environ Contam Toxicol.

[CR19] Braune BM, Gaskin DE (1987). A mercury budget for the Bonaparte's Gull during autumn moult. Ornis Scand.

[CR20] Braune BM, Noble DG (2009). Environmental contaminants in Canadian shorebirds. Environ Monit Assess.

[CR21] Braune BM, Scheuhammer AM (2008). Trace element and metallothionein concentrations in seabirds from the Canadian Arctic. Environ Toxicol Chem: an International Journal.

[CR22] Burgat V (1990). Un micropollutant: le Cadmium. Bulletin Mensuel de l’Office National de la Chasse 146, 40–42. ISSN: 0151–4806

[CR23] Burger J (1993). Metals in avian feathers: bioindicators of environmental pollution. Rev Environ Toxicol.

[CR24] Burger J (1996). Heavy metal and selenium levels in feathers of Franklin’s gulls in interior North America. Auk.

[CR25] Burger J, Gochfeld M (1985). Comparisons of nine heavy metals in salt gland and liver of Greater Scaup (*Aythya marila*), Black Duck (*Anas rubripes*), and Mallard (*A. platyrhynchos*). Compar Biochem Physiol.

[CR26] Burger J, Gochfeld M (1993). Lead and cadmium accumulation in eggs and fledgling seabirds in the New York Bight. Environ Toxicol and Chem.

[CR27] Burger J, Gochfeld M (2000). Metal levels in feathers of 12 species of seabirds from Midway Atoll in the northern Pacific Ocean. Sci Total Environ.

[CR28] Burger J, Nisbet ICT, Gochfeld M (1992). Metal levels in regrown feathers: assessment of contamination on the wintering and breeding grounds in the same individuals. J Toxicol Environ Health.

[CR29] Burger J, Gochfeld M, Sullivane K, Irons D, McKnightf A (2008). Arsenic, cadmium, chromium, lead, manganese, mercury, and selenium in feathers of Black-legged Kittiwake (*Rissa tridactyla*) and Black Oystercatcher (*Haematopus bachmani*) from Prince William Sound, Alaska. Sci Total Environ.

[CR30] Bustamante P, Caurant F, Fowler SW, Miramand P (1998). Cephalopods as a vector for the transfer of cadmium to top marine predators in the north-east Atlantic Ocean. Sci Total Environ.

[CR31] Clarke JU (1998). Evaluation of censored data methods to allow statistical comparisons among very small samples with below detection limit observations. Environ Sci Technol.

[CR32] Cobelo-Garcıa A, Prego R (2003). Heavy metal sedimentary record in a Galician Ria (NW Spain): background values and recent contamination. Mar Pollut Bull.

[CR33] Das K, Debacker V, Pillet S, Bouquegneau JM, Vos JG, Bossart G, Fournier M, O'Shea T (2003). Chapter 7: heavy metals in marine mammals. Toxicology of Marine Mammals.

[CR34] Dietz R, Riget F, Johansen P (1996). Lead, cadmium, mercury and selenium in Greenland marine animals. Sci Total Environ.

[CR35] Eagles-Smith CA, Suchanek TH, Colwell AE, Anderson NL, Moyle PB (2008). Changes in fish diets and food web mercury bioaccumulation induced by an invasive planktivorous fish. Ecol Appl.

[CR36] Egwumah FA, Egwumah PO, Edet DI (2017). Paramount roles of wild birds as bioindicators of contamination. Int J Avian & Wildlife Biol.

[CR37] Eisler R (2000). Handbook of chemical risk assessment: health hazards to humans, plants and animals.

[CR38] Elliott J, Scheuhammer A, Leighton F, Pearce P (1992). Heavy metal and metallothionein concentrations in Atlantic Canadian seabirds. Arch Environ Contam Toxicol.

[CR39] Fimreite N (1974). Mercury contamination of aquatic birds in northwestern Ontario. Journal of Wildlife Management C.

[CR40] Fowler SW (1990). Critical review of selected heavy metal and chlorinated hydrocarbon concentrations in the marine environment. Mar Environ Res.

[CR41] Frantz A, Federici P, Legoupi J, Jacquin L, Gasparini J (2016). Sex-associated differences in trace metals concentrations in and on the plumage of a common urban bird species. Ecotoxicol.

[CR42] Friberg L, Piscator M, Nordberg G, Kjellstrom T (1974). Cadmium in the environment II.

[CR43] Fu J, Wang Q, Wang H, Yu H (2014). Monitoring of non-destructive sampling strategies to assess the exposure of avian species in Jiangsu Province, China to heavy metals. Environ Sci Pollut Res.

[CR44] Geroudet P (1984). Origine mediterranéenne confirmée pour les Goélands leucophées du Léman. Nos Oiseaux.

[CR45] Gochfeld M (1997). Spatial patterns in a bioindicator: heavy metal and selenium concentration in eggs of Herring gulls (*Larus argentatus*) in the New York Bight. Arch Environ Contam Toxicol.

[CR46] Gochfeld M, Belant JL, Shukla T, Benson T, Burger J (1996). Heavy metals in laughing gulls: gender, age and tissue differences. Environ Toxicol Chem.

[CR47] Guideline IHT (2005). Validation of analytical procedures: text and methodology Q2 (R1). In International Conference on Harmonization, Geneva, Switzerland (pp. 11–12).

[CR48] Heinz GH (1996). Selenium in birds. In: Beyer WN, Heinz GH, Redmon-Norwood AW (eds) Environmental contaminants in wildlife: interpreting tissue concentrations. SETAC CRC, Lewis Boca Raton, FL, 447–458.

[CR49] Hernández-Moreno D, Ramos A, Romay CD, Fidalgo LE, Menozzi A, Bertini S (2021). Heavy metals content in great shearwater (*Ardenna Gravis*): accumulation, distribution and biomarkers of effect in different tissues. Arch Environ Contam Toxicol.

[CR50] Hoffman DJ (2002). Role of selenium toxicity and oxidative stress in aquatic birds. Aquat Toxicol.

[CR51] Honda K, Yamamoto Y, Hidaka H, Tatsukawa R (1986). Heavy metal accumulations in Adelin penguin *Pygoscelis adeliae*, and their variations with the reproductive processes. Mem Natl Inst Polar Res.

[CR52] Hutton M (1981). Accumulation of heavy metals and selenium in three seabird species from the United Kingdom. Environ Pollut Ser A.

[CR53] Kehrig HA, Hauser-Davis RA, Seixas TG, Fillmann G (2015). Trace elements, methylmercury and metallothionein levels in Magellanic penguin (*Spheniscus magellanicus*) found stranded on the Southern Brazilian coast. Mar Pollut Bull.

[CR54] Kim EY, Ichihashi H, Saeki K, Atrashkevich G, Tanabe S, Tatsukawa R (1996). Metal accumulation in tissues of seabirds from Chaun, northeast Siberia, Russia. Environ Pollut.

[CR55] Kim J, Oh JM (2017). Concentrations of Trace Elements in Adult and Nestling Black-Tailed Gulls (*Larus crassirostris*). Bull Environ Contam Toxicol.

[CR56] Koeman JH, Peeters WH, Koudstaal-Hol CH, Tijoe PS, Goeij JJ (1973). Mercury selenium correlations in marine mammals. Nature.

[CR57] Kojadinovic J, Le Corre M, Cosson RP, Bustamante P (2007). Trace elements in three marine birds breeding on Reunion Island (Western Indian Ocean) part 1: factors influencing their bioaccumulation. Arch Environ Contam Toxicol.

[CR58] Korbecki J, Gutowska I, Chlubek D, Baranowska-Bosiacka I (2019). Lead (Pb) in the tissues of Anatidae, Ardeidae, Sternidae and Laridae of the Northern Hemisphere: a review of environmental studies. Environ Sci Pollut Res Int.

[CR59] Koster MD, Ryckman DP, Weseloh DVC, Struger J (1996). Mercury levels in Great Lakes herring gull (*Larus argentatus*) eggs, 1972–1992. Environ Pollut.

[CR60] Kowal NE, Johnson DE, Kraemer DF, Pahren HR (1979). Normal levels of cadmium in diet, urine, blood, and tissues of inhabitants of the United States. J Toxicol Environ Health, Part A Current Issues.

[CR61] Kunito T, Kubota R, Fujihara J, Agusa T, Tanabe S (2008). Arsenic in marine mammals, seabirds, and sea turtles. In Reviews of environmental contamination and toxicology (pp. 31–69). Springer, New York, NY. 10.1007/978-0-387-77030-7_2 18418953

[CR62] Leonzio C, Fossi C, Focardi S (1986). Lead, mercury, cadmium and selenium in two species of gull feeding on inland dumps, and in marine areas. Sci Total Environ.

[CR63] Lewis SA, Furness RW (1991). Mercury accumulation and excretion in laboratory reared black-headed gull *Larus ridibundus* chicks. Arch Environ Contam Toxicol.

[CR64] Lindberg P, Odsjo T (1983). Mercury levels in feathers of Peregrine Falcon *Falco peregrinus* compared with total mercury content in some of its prey species in Sweden. Environ Pollut.

[CR65] Lucia M, Andre JM, Bernadet MD, Gontier K, Guy G, Davail S (2008). Concentrations of metals (zinc, copper, cadmium, and mercury) in three domestic ducks in France: Pekin, Muscovy, and Mule ducks. J Agric Food Chem.

[CR66] Majidi Y, Bahramifar N, Ghasempouri SM (2015). Pattern of mercury accumulation in different tissues of migratory and resident birds: Western reef heron (*Egretta gularis*) and Siberian gull (*Larus heuglini*) in Hara International Wetland-Persian Gulf. Environ Monit Assess.

[CR67] Mansouri B, Pourkhabbaz A, Babaei H, Hoshyari E (2012). Heavy metal contamination in feathers of Western Reef Heron (Egretta gularis) and Siberian gull (*Larus heuglini*) from Hara biosphere reserve of Southern Iran. Environ Monit Assess.

[CR68] Marier JR, Jaworski JF (1983). Interactions of selenium (Vol. 74). National Research Council Canada, Associate Committee on Scientific Criteria for Environmental Quality.

[CR69] Martin AJ, Lorenzo AJ (2001) Aves del archipiélago canario. Francisco Lemus, Editor. Tenerife, pp 787

[CR70] Mendes P, Eira C, Torres J, Soares AMVM, Melo P, Vingada J (2008). Toxic element concentration in the Atlantic gannet *Morus bassanus* (Pelecaniformes, Sulidae) in Portugal. Arch Environ Contam Toxicol.

[CR71] Méndez A, Montalvo T, Aymí R, Carmona M, Figuerola J, Navarro J (2020). Adapting to urban ecosystems: unravelling the foraging ecology of an opportunistic predator living in cities. Urban Ecosyst.

[CR72] Merian E (1991) Metals and their compounds in the environment: occurrence, analysis and biological relevance. VCH Verlagsgesellschaft mbH Weinheim (Germany)

[CR73] Migula P, Augustyniak M, Szymczyk A, Kowalczyk K (2000). Heavy metals, resting metabolism rates and breeding parameters in two populations of black-headed Gull *Larus ridibundus* from the industrially polluted areas of Upper Silesia, Poland. Acta Ornithol.

[CR74] Nardiello V, Fidalgo LE, López-Beceiro A, Bertero A, Martínez-Morcillo S, Míguez MP, Soler F, Caloni F, Pérez-López M (2019). Metal content in the liver, kidney, and feathers of Northern gannets, *Morus bassanus*, sampled on the Spanish coast. Environ Sci Pollut Res.

[CR75] Navarro G, Jerez S, Farinós P, Robledano F, Motas M (2010). Evaluación de la exposición a elementos inorgánicos (Cr, Mn, Ni, Cu, Zn, As, Se, Cd y Pb) en cormoranes grandes (*Phalacrocorax carbo* sinensis) de la laguna costera del Mar Menor de Murcia. Anales De Veterinaria Murcia.

[CR76] Neff JM (1997). Ecotoxicology of arsenic in the marine environment. Environ Toxicol Chem.

[CR77] Nicholson JK (1981). The comparative distribution of zinc, cadmium and mercury in selected tissues of the herring gull (*Larus argentatus*). Comp Biochem Physiol C Comp Pharmacol.

[CR78] Nielsen CO, Dietz R (1989). Heavy metals in Greenland seabirds. Commission for Scientific Research in Greenland.

[CR79] Ohlendorf HM, Kilness AW, Simmons JL, Stroud RK, Hoffman DJ, Moore JF (1988). Selenium toxicosis in wild aquatic birds. J Toxicol Envirom Health.

[CR80] Ohlendorf HM, Hothem RL, Welsh D (1989). Nest success, cause-specific nest failure, and hatchability of aquatic birds at selenium-contaminated Kesterson Reservoir and a reference site. Condor.

[CR81] Orlowski G, Polechonski R, Dobicki W, Zawada Z (2007). Heavy metal concentrations in the tissues of the Black-headed Gull Larus ridibundus L. nesting in the dam reservoir in south- western Poland. Pol J Ecol.

[CR82] Otero XL, de la Peña-Lastra S, Romero D, Nobrega GN, Ferreira TO, Pérez-Alberti A (2018). Trace elements in biomaterials and soils from a yellow-legged gull (*Larus michahellis*) colony in the Atlantic Islands of Galicia National Park (NW Spain). Mar Pollut Bull.

[CR83] Pain D (1987). Lead poisoning in waterfowl: an investigation of sources and screening techniques.

[CR84] Pain DJ, Sears J, Newton T (1995). Lead concentrations in birds of prey in Britain. Environ Pollut.

[CR85] Pařízek J, Oštádalová I, Kalousková J, Babický A, Pavlík L, Bíbr B (1971). Effect of mercuric compounds on the maternal transmission of selenium in the pregnant and lactating rat. Reproduction.

[CR86] Perco F, Leonzio C, Focardi S, Fossi C, Renzoni A (1983). Intossicazione da piombo in due Cigni reali della laguna d i Marano (nord-est Italia). Avocetta.

[CR87] Ramos R, Ramírez F, Jover L (2013). Trophodynamics of inorganic pollutants in a wide-range feeder: the relevance of dietary inputs and biomagnification in the yellow-legged gull (*Larus michahellis*). Environ Pollut.

[CR88] Sandersons G, Bellrose F (1986) A review of the problems of lead poisoning in waterfowl. Natural History Survey Special Publications 4

[CR89] Sarkka J, Hattula L, Paasivirta J, Janatuinen J (1978). Mercury and chlorinated hydrocarbons in the food chain of lake Paijanne, Finland. Holarctic Ecol.

[CR90] Savinov V, Gabrielsen G, Savinova T (2003). Cadmium, zinc, copper, arsenic, selenium and mercury in seabirds from the Barents Sea: levels, inter-specific and geographical differences. Sci Total Environ.

[CR91] Scheuhammer AM (1987). The chronic toxicity of aluminium, cadmium, mercury, and lead in birds: a review. Environ Pollut.

[CR92] SEO (2018). https://www.seo.org/ave/gaviota-patiamarilla/

[CR93] Stewart F, Furness R, Monteiro L (1996). Relationships between heavy metal and metallothionein concentrations in Lesser Black- Backed Gulls, *Larus fuscus*, and Cory’s Shearwater, *Calonectris diomedea*. Arch Environ Contam Toxicol.

[CR94] Stock MR, Herber FM, Geron MA (1989). Cadmium levels in oystercatcher *Haematopus ostralegus* from the German Wadden Sea. Mar Ecol Prog Ser.

[CR95] Vizuete J, Pérez-López M, Míguez-Santiyán MP, Hernández-Moreno D (2019). Mercury (Hg), lead (Pb), cadmium (Cd), selenium (Se) and arsenic (As) in liver, kidney and feathers of gulls: A review. Rev Environ Contam Toxicol.

[CR96] Watson P (1981). Seabird observations from commercial trawlers in the Irish Sea. British Birds C.

[CR97] Work TM, Smith MR (1996). Lead exposure in Laysan Albatross adults and chicks in Hawaii: prevalence, risk factors, and biochemical effects. Arch Environ Contain Toxicol.

[CR98] Yang DY, Chen YW, Gunn JM, Belzile N (2008). Selenium and mercury in organisms: interactions and mechanisms. Environ Rev.

[CR99] Zhang W, Ma J (2011). Waterbirds as bioindicators of wetland heavy metal pollution. Procedia Environ Sci.

[CR100] Zwolak I (2020). The role of selenium in arsenic and cadmium toxicity: an updated review of scientific literature. Biol Trace Elem Res.

