# Supplementary material for: Heavy metals and metalloid levels in the tissues of yellow-legged gulls (Larus michahellis) from Spain: sex, age, and geographical location differences
Source: Environ Sci Pollut Res Int. 2022 Mar 17;29(36):54292–308. doi: 10.1007/s11356-022-19627-8 (PMC9356949; doi:10.1007/s11356-022-19627-8)
Supplement: Supplementary file 1 — Supplementary file1 (DOCX 686 KB) [file 11356_2022_19627_MOESM1_ESM.docx]

**Heavy metals and metalloid levels in the tissues of yellow-legged gulls (*Larus michahellis*) from Spain: sex, age and geographical location differences.**

Jorge Vizuete^a^, David Hernández-Moreno^b,⁎^, Ana López-Beceiro^c^, Luis Eusebio Fidalgo^c^, Francisco Soler^a,d^, Marcos Pérez-López^a,e^, María Prado Míguez-Santiyán^a,e,⁎⁎^

^a^ Toxicology Area, Faculty of Veterinary Medicine (UEX), 10003 Caceres, Spain

^b^ National Institute of Agriculture and Food Research and Technology (INIA), Spanish National Research Council (CSIC), Department of Environment and Agronomy, Carretera de la Coruña Km 7 Madrid, Spain.

^c^ Department of Veterinary Clinical Sciences, Faculty of Veterinary Medicine (USC), 27003 Lugo, Spain

^d^ IMPROCAR Research Institutes, Spain

^e^ INBIO G+C Research Institutes, Spain

María Prado Míguez-Santiyán and David Hernández-Moreno contributed equally to this work.

⁎ Corresponding author at: National Institute of Agriculture and Food Research and Technology (INIA), Spanish National Research Council (CSIC), Department of Environment and Agronomy, Carretera de la Coruña Km 7 Madrid, Spain.

⁎⁎ Corresponding author at: Toxicology Area, Faculty of Veterinary Medicine (UEX), 10003 Caceres, Spain.

E-mail addresses: [Hernandez.david@inia.es](mailto:Hernandez.david@inia.es) (D. Hernández-Moreno), [prado.miguez@gmail.com](mailto:prado.miguez@gmail.com) (M.P. Míguez Santiyán).


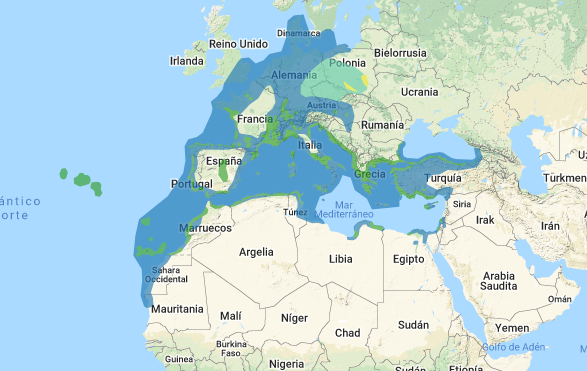

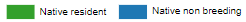

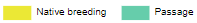


***Figure S1. Distribution map of L. michahellis. (Birdlife International, 2021)***

***Table S1. General conditions for the microwave digestion.***

| **Time (min)** | **Temperature (^o^C)** | **Potency (W)** | **Pressure (bar)** |
| --- | --- | --- | --- |
| 5 | 100 | 1200 | 70 |
| 7 | 130 | 1300 | 100 |
| 8 | 170 | 1300 | 120 |
| 10 | 200 | 1500 | 120 |
| 15 | 200 | 1400 | 120 |

***Table S2. Operation conditions for the ICP-MS analysis.***

| Potency RF (W) | 1550 |
| --- | --- |
| Plasma Mode | General purpose |
| Omega Bias (V) | -120 |
| Omega lens (V) | 9.3 |
| Extract 2 (V) | -245 |
| Deflect Lens (V) | 1.0 |
| Energy discrimination (V) | 5 |
| Collision gas (ml/min) | 5 |
| Cell Entrance (V) | -40 |
| Cell Exit (V) | -60 |
